# A phase II trial of personalized peptide vaccination in castration-resistant prostate cancer patients: prolongation of prostate-specific antigen doubling time

**DOI:** 10.1186/1471-2407-13-613

**Published:** 2013-12-30

**Authors:** Masanori Noguchi, Fukuko Moriya, Shigetaka Suekane, Rei Ohnishi, Satoko Matsueda, Tetsuro Sasada, Akira Yamada, Kyogo Itoh

**Affiliations:** 1 Clinical Research Division of the Research Center for Innovative Cancer Therapy, Kurume University School of Medicine, 67 Asahi-machi, Kurume 830-0011, Japan; 2 Departments of Urology, Kurume University School of Medicine, Kurume, Japan; 3 Immunology and Immunotherapy, Kurume University School of Medicine, Kurume, Japan; 4 Cancer Vaccine of the Research Center for Innovative Cancer Therapy, Kurume University School of Medicine, Kurume, Japan

**Keywords:** Prostate-specific antigen doubling time, Personalized peptide vaccine, Prostate cancer, Surrogate marker, Overall survival

## Abstract

**Background:**

Cancer vaccine is one of the attractive treatment modalities for patients with castration-resistant prostate cancer (CRPC). However, because of delayed immune responses, its clinical benefits, besides for overall survival (OS), are not well captured by the World Health Organization (WHO) and Response Evaluation Criteria in Solid Tumors (RECIST) criteria. Several surrogate markers for evaluation of cancer vaccine, including prostate-specific antigen doubling time (PSADT), are currently sought. The purpose of this study was to assess prospectively the PSA kinetics and immune responses, as well as the efficacy, safety, and biomarkers of personalized peptide vaccination (PPV) in progressive CRPC.

**Methods:**

One hundred patients with progressive CRPC were treated with PPV using 2–4 positive peptides from 31 candidate peptides determined by both human leukocyte antigen (HLA) class IA types and the levels of immunoglobulin G (IgG) against each peptide. The association between immune responses and PSADT as well as overall survival (OS) was studied.

**Results:**

PPV was safe and well tolerated in all patients with a median survival time of 18.8 months. Peptide-specific IgG and T-cell responses strongly correlated with PSADT (*p* < 0.0001 and *p* = 0.0007, respectively), which in turn showed correlation with OS (*p* = 0.018). Positive IgG responses and prolongation of PSADT during PPV were also significantly associated with OS (*p* = 0.001 and *p* = 0.004) by multivariate analysis.

**Conclusions:**

PSADT could be an appropriate surrogate marker for evaluation of the clinical benefit of cancer vaccine. Further randomized trials are needed to confirm these results.

**Trial registration:**

UMIN000001850

## Background

Changes in serum prostate-specific antigen (PSA) can reflect the burden of disease and clinical benefit in patients with castration-resistant prostate cancer (CRPC) with cytotoxic chemotherapy or hormonal agents known to kill tumor cells; these changes can have practical utility by providing and updating prognostic information on an individual patient over time [1-4]. As observed in many clinical trials, however, immunotherapy can induce novel patterns of antitumor responses distinct from those of chemotherapy [5]. For example, an autologous dendritic-cell-based vaccine (sipuleucel-T) is known to improve survival without having an impact on early PSA decline [6], whereas docetaxel's improvement in overall survival (OS) correlates for the most part with a PSA decline within the first 3 months of therapy [7,8]. Thus, interpreting PSA decline in the context of novel immunotherapy must be carried out with caution on the basis of the mechanism of action, and may also depend on the time of sampling [9].

Personalized peptide vaccine (PPV) uses multiple peptides based on the pre-existing immunity. Under PPV treatment, each patient with human leukocyte antigen (HLA)-class IA types positive was tested for their immunological reactivity to 31 different peptides capable of inducing T-cell responses. The 31 peptides were derived from a number of tumor associated antigens: PSA, prostatic acid phosphatase (PAP), prostate-specific membrane antigen (PSMA), multidrug resistance protein and a variety of other epithelial tumor antigens. We previously demonstrated that PPV was safe and improved OS with immune responses in phase I, I/II, and II clinical trials in patients with CRPC [10-16]. However, it was not addressed whether PSADT could be an appropriate surrogate marker for evaluation of the clinical benefit of cancer vaccine. To address this, we evaluated data from a phase II clinical trial for CRPC using PPV.

## Methods

### Patient Eligibility

Eligibility required a histological diagnosis of prostate adenocarcinoma and progressive disease (PD) defined as at least two consecutive increases in PSA, new metastatic lesion on radionuclide bone scan, or progressive tumor lesions on cross-sectional imaging, despite adequate androgen ablative therapy. Patients showed positive IgG responses to at least two of the 31 different candidate peptides (Table 1). Any number of previous hormonal therapies was allowed. Patients were required to wait at least four weeks for entry into the study after the completion of prior radiation therapy, chemotherapy, or a change in hormonal therapy. Other inclusion criteria included age ≥ 20 years; Eastern Cooperative Oncology Group (ECOG) performance status 0 or 1; life expectancy of at least 12 weeks; positive status for HLA-A2, -A24, -A3 supertype (−A3, -A11, -A31, and -A33), or -A26; adequate hematologic, hepatic, and renal function; and negative status for hepatitis virus B and C. Exclusion criteria included an acute infection; a history of severe allergic reactions; pulmonary, cardiac, or other systemic diseases; and other inappropriate conditions for enrollment as judged by clinicians.

**Table 1:** Peptide candidates for personalized peptide vaccination

**Symbol for peptide**	**Origin protein**	**Position of peptide**	**Amino acid sequence**	**HLA type**
CypB-129	Cyclophilin B	129-138	V	A2,A3sup^a^
Lck-246	p56 lck	246-254	KLVERLGAA	A2
Lck-422	p56 lck	422-430	DVWSFGILL	A2,A3sup
MAP-432	ppMAPkkk	432-440	DLLSHAFFA	A2,A26
WHSC2-103	WHSC2	103-111	ASLDSDPWV	A2,A3sup^a^,A26
HNRPL-501	HNRPL	501-510	NVLHFFNAPL	A2,A26
UBE-43	UBE2V	43-51	RLQEWCSVI	A2
UBE-85	UBE2V	85-93	LIADFLSGL	A2
WHSC2-141	WHSC2	141-149	ILGELREKV	A2
HNRPL-140	HNRPL	140-148	ALVEFEDVL	A2
SART3-302	SART3	302-317	LLQAEAPRL	A2
SART3-309	SART3	309-317	RLAEYQAYI	A2
SART2-93	SART2	93-101	DYSARWNEI	A24
SART3-109	SART3	109-118	VYDYNCHVDL	A24,A3sup^a^,A26
Lck-208	p56 lck	208-216	HYTNASDGL	A24
PAP-213	PAP	213-221	LYCESVHNF	A24
PSA-248	PSA	248-257	HYRKWIKDTI	A24
EGFR-800	EGF-R	800-809	DYVREHKDNI	A24
MRP3-503	MRP3	503-511	LYAWEPSFL	A24
MRP3-1293	MRP3	1293-1302	NYSVRYRPGL	A24
SART2-161	SART2	161-169	AYDFLYNYL	A24
Lck-486	p56 lck	486-494	TFDYLRSVL	A24
Lck-488	p56 lck	488-497	DYLRSVLEDF	A24
PSMA-624	PSMA	624-632	TYSVSFDSL	A24
EZH2-735	EZH2	735-743	KYVGIEREM	A24
PTHrP-102	PTHrP	102-111	RYLTQETNKV	A24
SART3-511	SART3	511-519	WLEYYNLER	A3sup^a^
SART3-734	SART3	734-742	QIRPIFSNR	A3sup^a^
Lck-90	p56 lck	90-99	ILEQSGEWWK	A3sup^a^
Lck-449	p56 lck	449-458	VIQNLERGYR	A3sup^a^
PAP-248	PAP	248-257	GIHKQKEKSR	A3sup^a^

^a^A3sup, HLA-A3 supertype (A3, A11, A31, and A33).

### Study design and treatment

This was a single institution, single arm, open-label, phase II study. The endpoints of this study were primarily safety and feasibility of PPV in patients with CRPC. Secondary endpoints were to assess the PSA kinetics and immune responses. In addition, we identified potential factors for predicting OS and selecting suitable patients for this treatment. This study protocol was approved by Kurume University Ethical Committee. Written informed consent was obtained from all patients before any study procedures.

In this study, 31 peptides, whose safety and immunological effects had been confirmed in previously conducted clinical studies [10-18], were employed for vaccination [12 peptides for HLA-A2, 14 peptides for HLA-A24, 9 peptides for the HLA-A3 supertype (A3, A11, A31, or A33), and 4 peptides for HLA-A26] (Table 1). All peptides were prepared under conditions of Good Manufacturing Practice using a Multiple Peptide System (San Diego, CA). The selection of 2 to 4 peptides for vaccination to each patient was based on HLA typing and high titer level of peptide-specific IgG to candidate peptides. Each of the selected peptides was mixed with incomplete Freund’s adjuvant (Montanide ISA-51VG; Seppic, Paris, France) and emulsified in the 5 ml plastic syringe, and a maximum of four peptides of 1.5 ml emulsion (3 mg/peptide) were injected subcutaneously into the lateral thigh area once a week for 6 weeks. The peptides were re-selected according to peptide-specific IgG levels at every cycle of 6 vaccinations and administered at 2-, 3-, or 4-week intervals until withdrawal of consent or unacceptable toxicity.

### Assessment of clinical activity

Patients were monitored at each visit by history and physical examinations. Serum PSA test and routine laboratory studies were performed every 6 vaccinations for any adverse effects. Toxicity was graded according to the National Cancer Institute Common Terminology Criteria for Adverse Events version 3.0 (NCI-CTCAE Ver3).

All patients underwent relevant radiologic studies and bone scans every 6 months or at the progression of symptoms. PD was defined as radiographic progression evaluated by Response Evaluation Criteria in Solid Tumors (RECIST) criteria [19] or clinical progression.

To assess the PSA response for each patient, percent PSA change from baseline was calculated for each phase of the study (pre- and during vaccination). In addition, PSA doubling time (PSADT) was calculated using all serum PSA values for a specified period, and using a minimum of three PSA values by the formula log_2_/b, where b denotes the least square estimate of the linear regression model of the log-transformed PSA values on time. For analytical purposes, negative PSADT estimates and high positive PSADT estimates (>36 months) were censored at 36 months.

To investigate biomarkers for OS that may allow patient selection and prediction of a response to PPV, serum amyloid A (SAA), C-reactive protein (CRP), and interleukin (IL)-6 in plasma at baseline were additionally examined by enzyme-linked immunosorbent assay (ELISA), respectively.

### Measurement of humoral and T-cell responses specific to the vaccinated peptides

To study the humoral responses specific to the vaccinated peptides, peptide-specific IgG levels were measured by a Luminex system (Luminex, Austin, TX), as reported previously [20]. If the total titers of selected peptide-specific IgG in any cycles of post-vaccination plasma were more than 2-fold higher than those in the pre-vaccination plasma, the changes were considered to be a positive response.

Although T-cell subsets using flowcytometry was not analyzed in this study, T-cell responses specific to the vaccinated peptides were evaluated by IFN-γ ELISPOT assay using peripheral blood mononuclear cells (PBMCs), as reported previously [18]. Peptide-specific T-cell responses were evaluated by the differences between the numbers of spots per 10^5^ x PBMCs in response to the vaccine peptides and those to the control peptide at pre- and 6th vaccination; at least 2-fold more spots at the 6th vaccination than at pre-vaccination was considered positive.

### Statistical analysis

All patients who received more than 6 vaccinations were considered evaluable for tumor response, and all patients entered were included in the survival analysis. Data were analyzed at the end of November, 2012 using commercially available computer software. The Student’s t-test and the chi-square test were used to compare quantitative and categorical variables, respectively. Survival was calculated from the date of first treatment until the date of any cause of death. Patients lost to follow-up were censored at the last known date of survival. The Kaplan-Meier method was used to estimate actuarial survival curves, and groups were compared using a log-rank test. Cox proportional hazards regression model was used for univariate and multivariate analyses to identify factors that had a significant impact on survival. All baseline parameters in the survival and proportional hazards regression analysis were analyzed as dichotomous variables using median or cut-off values. A two-sided significance level of 5% was considered statistically significant.

## Results

### Characteristics of the patients

Between April 2009 and August 2011, 100 patients with CRPC were enrolled in this trial at Kurume University Hospital. All 100 patients received at least one vaccination with a median of 16 vaccinations (range, 1 to 40) and were included in the safety assessment and survival analysis. Three patients did not complete 6 vaccinations (1 cycle) and were excluded from the assessment of PSA response and immune responses. The reason for these failures to complete 6 vaccinations was withdrawal of consent. The median age of participants was 69 years (range, 51 to 92 years), and the ECOG performance status was 0 in 91of the patients and 1 in the remaining 9. The median PSA and pre-vaccination PSADT at the entry to the study was 29.8 ng/ml (range, 0.2 to 2481 ng/ml) and 2 months (range, 0.3 to 36+ months), respectively. Fifty-seven patients had a Gleason score of ≥ 8 and 86 patients had metastasis. All patients had experienced progression after androgen deprivation therapy as an initial or secondary therapy. Forty patients had received docetaxel based chemotherapy with a median cycle of 6.5 as a third line treatment. Baseline patient characteristics are shown in Table 2.

**Table 2:** Patient characteristics

	**Patients (N = 100)**	
**Characteristics**	**No.**	
Age, years		
Median		69
Range		51-92
ECOG performance status		
0	91	
1	9	
HLA typing		
A24	66	
A2	21	
A3 supertype	11	
A26	2	
Baseline PSA, ng/ml		
Median		29.8
Range		0.2-2481
PSADT, months		
Median		2
Range		0.3-36+
Lymphocyte, 1300/μL		
Low	41	
High	59	
CRP, 3 μg/mL		
Low	53	
High	47	
SAA, 8 μg/mL		
Low	27	
High	76	
IL6, 2.4 pg/mL		
Low	84	
High	16	
Gleason score		
≤7	34	
≥8	57	
Unknown	9	
Site of metastasis		
no	14	
Bone only	33	
Bone and nodal/organ	40	
Nodal/organ	13	
Prior chemotherapy		
(-)	60	
(+)	40	

*Abbreviations*: PPV, personalized peptide vaccination; ECOG, Eastern Cooperative Oncology Group; HLA, human leucocyte antigen; PSA, prostate-specific antigen; PSADT, PSA doubling time; CRP, C-reactive protein; SAA, serum amyloid A; IL6, interleukin 6.

### Adverse events

The overall toxicities are shown in Table 3. The most frequent adverse events were local redness and swelling at injection sites, bone pain, hypoalbuminemia, lymphocytopenia, appetite loss, fatigue, increased ALP, and anemia, which were grade 1 or 2 in most cases. There were no grade 4 toxicities and no treatment-related deaths. A total of 51 grade 3 toxicities including anemia, bone pain, increased ALP, lymphocytopenia, decreased white blood cells, increased creatinine, injection site reaction, and increased AST and ALT were observed during the study. All of these severe adverse events were concluded to be not directly associated with the vaccinations, but with cancer progression or other causes by the independent safety evaluation committee in this trial.

**Table 3:** Adverse events during peptide vaccination

	**Grade 1**	**Grade 2**	**Grade 3**	**Total**
Injection site reaction	73	24	13	43
Constitutional symptoms				
Bone pain	16	14	13	43
Appetite loss	29	5	1	35
Fatigue	23	11	0	34
Edema peripheral	10	3	0	10
Blood/bone marrow				
Lymphocytopenia	17	13	5	35
Anemia	7	7	16	30
White blood cell count decreased	6	6	5	17
Laboratory				
Hypoalbuminemia	27	13	0	40
ALP increased	20	8	6	34
AST increased	24	4	1	29
Hyponatremia	24	1	0	25
ALT increased	13	2	1	16
Blood triglycerides increased	10	2	0	12
Creatinine increased	6	1	2	8

### Clinical outcome

**Figure 1:**
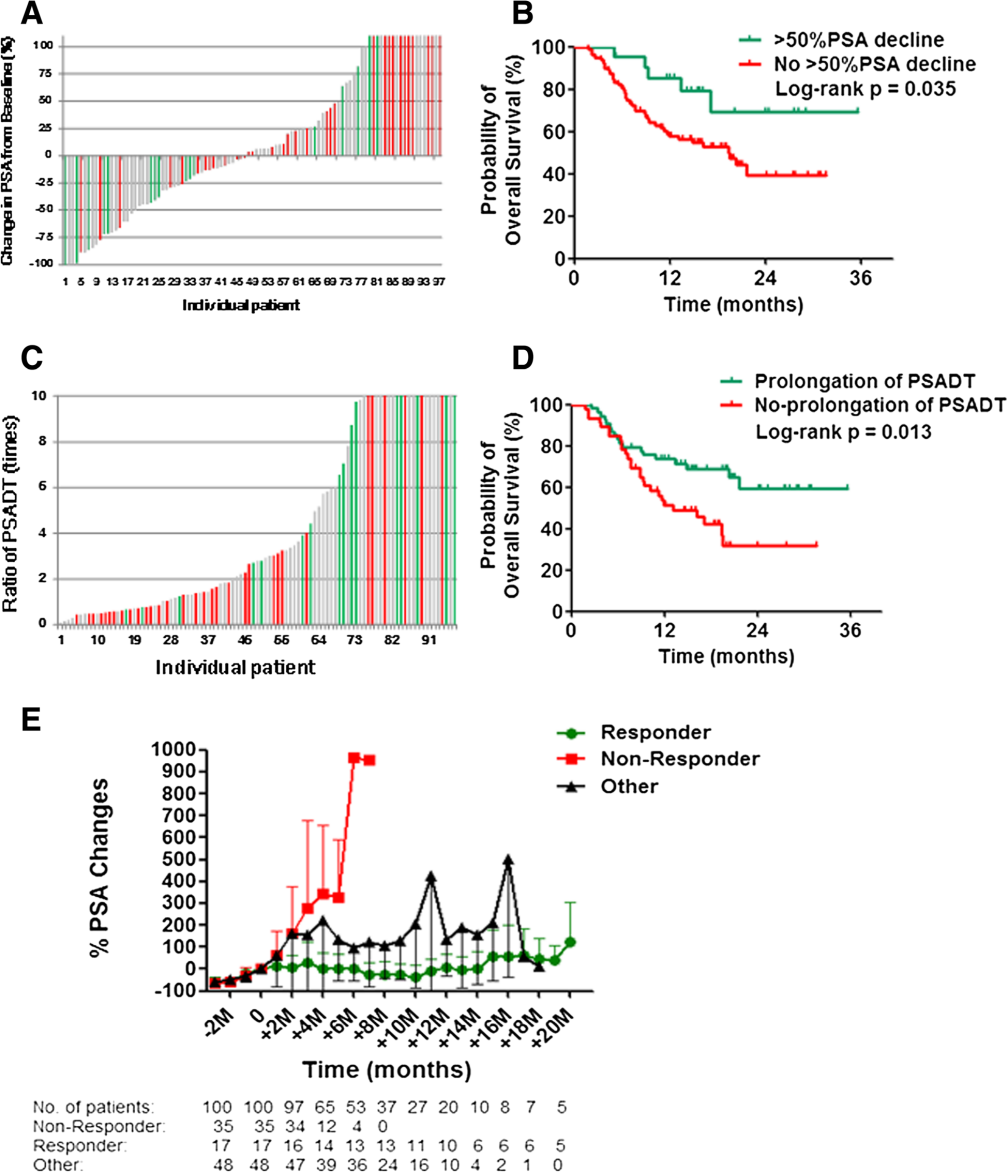
**PSA kinetics and overall survival. (A)** Waterfall plot showing the maximal PSA changes (%) from baseline during personalized peptide vaccination (PPV) at any time point. **(B)** Overall survival by >50% PSA decline. **(C)** The ratio of PSADT changes for each patient pre- and during PPV is plotted. The ratio of PSADT changes was calculated by dividing PSADT during treatment by pre-treatment PSADT. A ratio greater than 2 indicates prolongation of PSADT. **(D)** Overall survival by prolongation of PSDT. **(E)** Longitudinal average PSA changes (%) before and during PPV. Green histograms: Responder group (alive for more than 20 months). Red histograms: Non-responder group (death within 12 months). Gray histograms: Other group.

Forty-eight (49%) patients exhibited some decrease in PSA from baseline, ranging from 1.9% to 99.6% (Figure 1A). Confirmed ≥50% PSA decline at any point during PPV was observed in 21 patients (22%), with a median time of 4 months to ≥50% PSA decline and a median duration of ≥50% PSA decline of 3 months. Delayed PSA response was observed. Patients with ≥50% PSA decline during PPV showed longer survival than remaining patients ( *p* = 0.035) (Figure 1B). The median estimated PSADT pre- and during PPV were 2 and 3.89 months, respectively. Fifty-four (56%) patients displayed at least 2-fold increase over the pre-treatment PSADT (range, 2.1- to 75-fold), and these patients with a prolongation of PSADT showed longer survival than patients without a prolongation of PSADT (*p* = 0.013) (Figure 1C and D). To compare the difference in PSA responses with clinical outcomes, patients were divided into three groups: responder group with survival longer than 20 months after PPV, non-responder group with death within 12 months after PPV, and another group with the remaining patients. Average% PSA changes in the responder group were significantly lower than those in the non-responder group at 2 to 5 months (*p* < 0.005) and those in the other group at 5 to 10 months (*p* < 0.005) during the PPV. In addition, average% PSA changes in the responder group showed a trend of PSA plateau. Average% PSA changes from baseline among three groups before and during PPV are shown in Figure 1E.

There was no complete response or partial response in terms of measurable disease. The median time to disease progression, as defined by clinical and/or radiologic criteria, was 10.9 months (95% CI, 6 to 19 months). At the time of analysis with a median follow-up of 18 months (95% CI, 14.1 to 24 months), 64 deaths had occurred. Median survival time was 18.8 months (95% CI, 14.9 to 28.6 months) in all patients. Median survival time in chemotherapy naive patients and in patients after docetaxel chemotherapy were 21.6 months and 11.6 months, respectively.

### Immunological response

The number of selected peptides were 4 peptides in 62 patients, 3 peptides in 17 patients and 2 peptides in 21 patients at the first screening. Same peptide at the first screening were only selected in 29 of 97 (30%) patients at second screening and in 10 of 66 (15%) patients at the third screening, remaining patients received at least 1 different peptide during the study. The most frequently selected peptides were Lck486 (40 patients), CypB129 (31 patients), PAP213 (24 patients), SART2-93 (21 patients), PSA248 (20 patients), Lck488 (17 patients) and WHSC2-123 (16 patients) at the first screening. All 31 peptides were selected at any screening in the study.

**Figure 2:**
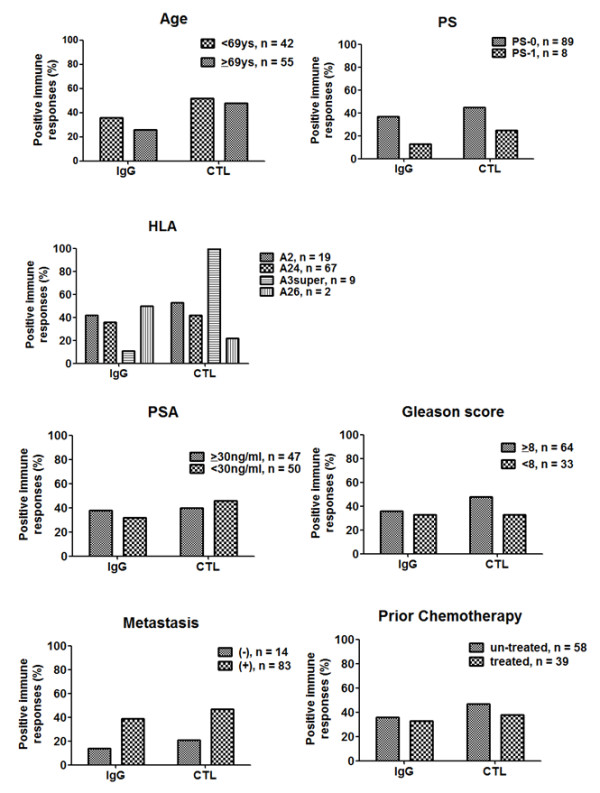
Positive immune responses of IgG and CTL based on baseline characteristics.

**Figure 3:**
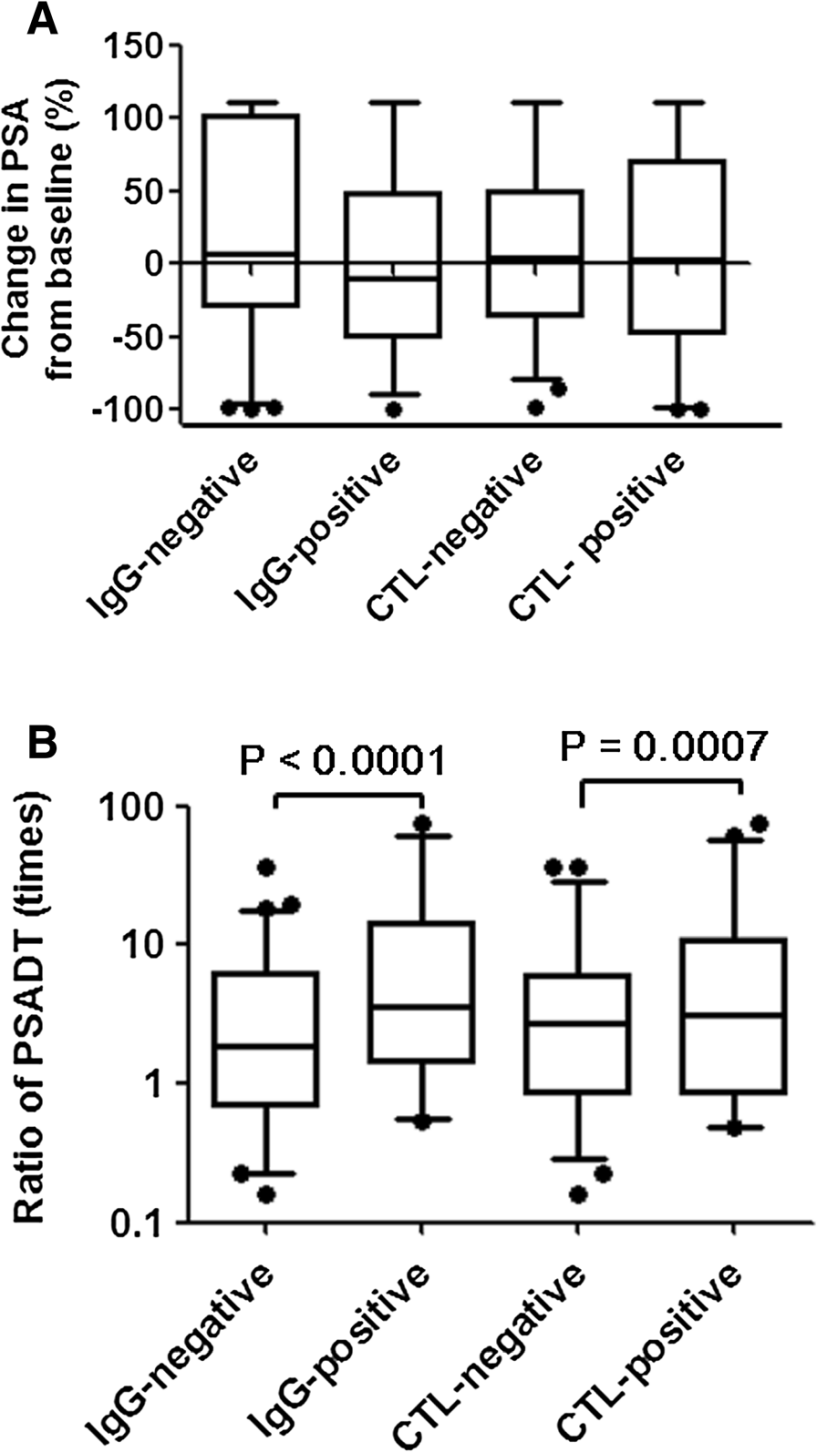
**Comparing immune responses with PSA kinetics. (A)** Change in PSA from baseline (%) based on immune responses. **(B)** Ratio of PSADT based on immune responses.

Total IgG responses specific to the vaccinated peptide were augmented in 42 of 97 (43%) patients, 62 of 66 (94%) patients, 36 of 36 (100%) patients, 16 of 16 (100%) patients, and 7 of 7 (100%) patients at the 6th, 12th, 18th, 24th, and 30th vaccinations, respectively. Finally, positive IgG responses during PPV were observed in 76/97 (79%) patients. PBMCs from 97 patients were available for IFN-γ Elispot assay at the pre- and 6th vaccination. Peptide-specific T-cell responses were detectable in 42 patients (43%) at the 6th vaccination. There was no obvious correlation between IgG and CTL responses. Positive immune responses of both IgG and CTL based on baseline characteristics including age, PS, HLA typing, PSA, Gleason score, presence of metastasis and prior chemotherapy are shown in Figure 2. There was no difference in positive immune responses among baseline characteristics. In comparing immune responses with PSA kinetics, although average PSA changes did not correlate with immune responses, average ratio of PSADT was significantly higher in patients with positive IgG (8 vs. 4, *p* < 0.0001) and CTL (8.8 vs. 6.1, *p* = 0.0007) responses (Figure 3).

### Survival analysis

Cox proportional hazards regression analysis was performed to determine factors that would predict disease death (Table 4). Univariate Cox analysis showed that good performance status (*p* < 0.0001), positive IgG response (p < 0.0001), low CRP (*p* = 0.012), prolongation of PSADT (*p* = 0.018), low PSA (*p* = 0.004), prior chemotherapy status (*p* = 0.037), positive T-cell response (*p* = 0.039), and presentation of ≥50% PSA decline (*p* = 0.046) were significantly associated with survival.

**Table 4:** Cox proportional hazards regression analysis of association between potential factors and death after PPV in the 100 CRPC patients

**Factors**	**Cut-offs**^**a**^	**Univariate**			**Multivariate**		
		**p value**	**Hazard ratio**	**95% CI**	**p value**	**Hazard ratio**	**95% CI**
IgG response	Positive vs. negative	<0.0001	0.19	0.101-0.355	0.001	0.272	0.125-0.592
ECOG performance status	0 vs. 1	<0.0001	0.073	0.031-0.174	0.004	0.179	0.056-0.569
CRP	Low (<3000 ng/mL) vs. high	0.012	0.461	0.252-0.842	0.006	0.389	0.199-0.759
PSADT	Increase (2 times) vs. no	0.018	0.477	0.258-0.881	0.004	0.357	0.176-0.725
PSA	Low (<30 ng/mL) vs. high	0.004	0.407	0.221-0.749	0.008	0.361	0.171-0.762
Prior chemotherapy	Untreated vs. treated	0.037	0.536	0.298-0.962	0.329	0.695	0.335-1.445
T-cell response	Positive vs. negative	0.039	0.51	0.269-0.967	0.273	0.679	0.340-1.357
>50% PSA decline	Positive vs. negative	0.046	0.387	0.152-0.984	0.553	0.733	0.263-2.042
Number of lymphocytes	High (>1300/μL) vs. low	0.054	0.562	0.313-1.009	-	-	-
IL6	Low (<2.4 pg/mL) vs. high	0.057	0.491	0.236-1.021	-	-	-
Pts. age	Low (<69 years) vs. high	0.186	0.666	0.364-1.218	-	-	-
Gleason score	Low (<8) vs. high	0.623	1.162	0.637-2.128	-	-	-
SAA	Low (<8 μg/mL) vs. high	0.709	0.875	0.433-1.767	-	-	-

Of the 100 men, 64 died.

^a^Lymphocyte, PSA, and patient age are based on median values.

*Abbreviations*: PPV, personalized peptide vaccination; CRPC, castration-resistant prostate cancer; CI, confidence intervals; ECOG, Eastern Cooperative Oncology Group; PSA, prostate-specific antigen; PSADT, PSA doubling time; CRP, C-reactive protein; SAA, serum amyloid A; IL6, interleukin 6.

The factors showing *p* less than 0.05 in the univariate analysis were included in multivariate analysis of the model. Finally, positive IgG response (*p* = 0.001) and prolongation of PSADT (*p* = 0.004) during PPV, as well as baseline good performance status (*p* = 0.004), low CRP levels (*p* = 0.006), and low PSA levels (*p* = 0.008), were significantly favorable factors for OS (Table 4).

## Discussion

As observed in several clinical trials, immunotherapy can induce novel patterns of antitumor responses distinct from those of chemotherapy, which are consequently not captured by the WHO or RECIST criteria [5]. On the other hand, there is debate regarding the utility of PSA changes, especially with immunotherapy, and the PSA Working Group 2 has advocated using radiographic progression-free survival as a preferred endpoint for phase II trials [21]. Others have argued that changes in PSADT may be a marker of drug effect, understanding that shorter PSADT corresponds to worse prognosis and, thus, a favorable change in PSADT suggests drug activity [22,23]. However, clinical trials of recently developed drugs, such as sipuleucel-T [6], cabazitaxel [24], and abiraterone acetate [25], for the treatment of progressive CRPC patients did not analyze the usefulness of PSADT as a surrogate marker of response in CRPC patients. In the current study, we attempted careful and stringent collection of multiple PSA values in order to calculate PSADT changes before and during PPV accurately. While delayed PSA responses were observed, we did see a statistically significant increase in PSADT. Importantly, patients with prolongation of PSADT showed statistically longer survival (*p* = 0.018). These results suggest that the development of late immune responses is associated with changes in PSADT.

The evaluation of T-cell immune responses to target self antigens after vaccine clinical trials presents several challenges. Antigen-specific T-cells can be evaluated by their peptide target specificity, proliferative capacity, cytokine secretion, cytolytic activity, and membrane markers of activation. At present, the best measure of antigen-specific T-cells is unknown, as is the optimal time to evaluate immune responses. In our current analysis, we evaluated both humoral responses determined by peptide-specific IgG levels using a Luminex system and antigen-specific CD8+ T-cell responses by using IFN-γ ELISPOT assays, to provide a more direct quantitative assessment after immunization. Delayed 50% PSA decline and prolongation of PSADT were observed in patients with positive IgG and T-cell respkonses, and these immune responses were associated with OS. These results suggest that further immunological analysis at multiple time points might be needed to determine whether T-cell response or the development of late immune responses is associated with clinical responses.

Cancer vaccinations do not always extract good immune and/or clinical responses in vaccinated patients. This study showed that IgG responses and prolongation of PSADT during PPV, along with baseline performance status, CRP, and PSA levels, were well correlated with OS in patients with CRPC treated by PPV. These results suggest that risk stratification based on these factors could be helpful for estimating the OS in patients with CRPC treated by immunotherapy.

Despite these encouraging observations, the current study must be interpreted as hypothesis-generating due to several limitations. This single-arm phase II study without a concurrent control arm did not allow estimation of the potential clinical or immune effects of this treatment. Another potential limitation of this study regarding OS is the lack of treatment data after the treatment phase of the trial. Imbalances due to chance may have occurred in treatments after progression. However, only docetaxel has been shown to affect survival in this population of patients, and only by a few months. The median survival of 18.8 months (95% CI, 14.1 to 24 months) observed in this study surpassed the survival that was observed from docetaxel-based clinical trials in a similar population by TAX-327 (median survival, 19.2 months) and South West Oncology Group 9906 (median survival, 17.5 months) [7,8]. Thus, we think it unlikely that a potential imbalance in post-study treatments could explain the survival results.

## Conclusions

This study showed that PPV in patients with CRPC was active and well tolerated, improving survival with immune responses, delayed PSA responses, and prolongation of PSADT. Further randomized trials are needed to confirm these preliminary results.

## Abbreviations

CR: Complete response; CT: Computed tomography; CRPC: Castration-resistant prostate cancer; CTL: Cytotoxic T lymphocytes; EOCG: Eastern cooperative oncology group; HLA: Human leukocyte antigen; IFN- γ: Interferon-γ; IgG: Immunoglobulin G; OS: Overall survival; PBMC: Peripheral blood mononuclear cells; PPV: Personalized peptide vaccination; PSA: Prostate specific antigen; PSADT: Prostate specific antigen doubling time.

## Competing interests

K. Itoh is a consultant/advisory board member in Green Peptide Co. A.Yamada is a part-time executive of Green Peptide Co. No potential conflicts of interest were disclosed by other authors.

## Authors' contributions

NM conceived of the study, and participated in its design and coordination and drafted the manuscript. KI and AY participated in its design and helped to draft the manuscipt. FM, SS, RO performed the clinical trial and collected the data. SM and TS carried out the immunoassays. All authors read and approved the final manuscript.

## Pre-publication history

The pre-publication history for this paper can be accessed here:

http://www.biomedcentral.com/1471-2407/13/613/prepub
